# Students Teaching Students: Student-Led Ultrasound Curriculum in Medical School Education

**DOI:** 10.7759/cureus.19332

**Published:** 2021-11-07

**Authors:** Michelle K Oberoi, Niresh C Perera, Josue Reynaga, Bo Ram Yoo, Christopher C Miller, Wesley Lockhart, Mo Entezampour, Lucas Friedman

**Affiliations:** 1 Emergency Medicine, University of California, Riverside School of Medicine, Riverside, USA; 2 Emergency Medicine, Harbor–University of California Los Angeles Medical Center, Los Angeles, USA; 3 Family Medicine, Scripps Mercy Hospital Chula Vista, Chula Vista, USA; 4 Psychiatry, University of California Riverside, Riverside University Health System, Riverside, USA; 5 Medical Simulation and Research, University of California, Riverside School of Medicine, Riverside, USA

**Keywords:** simulation, self-directed learning, assessment, sim, ultrasonography, bedside ultrasound, pocus, point-of-care, education, ultrasound

## Abstract

Introduction

With the expanding use of point-of-care ultrasound throughout medical specialties for the rapid bedside assessment integral to patient care, medical schools have sought to incorporate ultrasound education into their curriculum. Second-year medical students (MS2s) at our institution met this demand by forming the Ultrasound Student Instructor Cadre (USSIC), a longitudinal ultrasound curriculum where MS2s teach first-year medical students (MS1s). The objectives of this study were to assess the ultrasound knowledge of medical students and their perceptions of ultrasound incorporation into their medical education.

Methods

Our flipped classroom curriculum consisted of four lessons (cardiopulmonary, gastrointestinal, genitourinary, and musculoskeletal) composed of videos, didactic lessons, and hands-on probe-time, with 15-minute pre- and post-assessments. Paired Wilcoxon signed-rank tests were performed to evaluate the differences in the pre- and post-assessment scores for each teaching session. Additionally, an end-of-the-year survey assessed the perceived preparedness and overall satisfaction of the MS1s with the course.

Results

The differences between the pre- and post-assessments for each teaching session were statistically significant: cardiopulmonary (45.6 ± 16.9% vs. 82.9 ± 9.4%, *p* < 0.0001, n = 55), gastrointestinal (53.9 ± 18.0% vs. 84.1 ± 13.5%, *p* < 0.0001, n = 54), genitourinary (68.9 ± 19.1% vs. 91.4 ± 14.4%, *p* < 0.0001, n = 64), and musculoskeletal (33.6 ± 14.7% vs. 78.2 ± 18.2%, *p* < 0.0001, n = 55).

Conclusion

Our study suggests that MS1s met the learning objective for each teaching session. Furthermore, MS1s who became USSIC instructors as MS2s felt more prepared and were more satisfied with the course. This study demonstrates the efficacy of student-led instruction in ultrasound, and we offer our model for adoption into other medical schools.

## Introduction

The use of point-of-care ultrasound (POCUS) has become routine in various specialties because of its rapid, non-invasive way to assess and diagnose patients. While many residency programs continue to integrate ultrasound training and some specialties, such as emergency medicine, anesthesia, and obstetrics and gynecology have previously required Accreditation Council for Graduate Medical Education (ACGME) ultrasound milestones, residents who had limited ultrasound exposure as medical students may have a resulting discrepancy in ultrasound proficiency [1-3]. Consequently, medical schools have started incorporating ultrasound early in their curriculum [4-6]. However, this often takes the form of instructional materials and lectures with inadequate hands-on exposure ultimately resulting in an insufficient foundation in ultrasound [1]. Introducing ultrasound training concurrently with traditional undergraduate medical education (UME) enhances the overall knowledge and clinical skills of the students [1,4-10]. While some medical schools have incorporated an ultrasound curriculum that only trains first-year medical students (MS1s), other schools have expanded its implementation into a two- to four-year vertical curriculum to longitudinally reinforce students’ skills beyond the first year [1].

Despite the increasing integration of ultrasound in patient care and medical education, there are barriers to implementing a structured ultrasound curriculum. In a questionnaire distributed to academic deans from 134 allopathic medical schools, 78.9% of respondents acknowledged that ultrasound was important and should be part of the UME and that the major barriers to implementation were the lack of space in the curriculum and the funding to maintain ultrasound equipment and support trained physician faculty to teach [11].

Considering these challenges and given the benefits of ultrasound training during UME and the specific long-term benefit for students going into primary care, our institution implemented a student-driven teaching model, now known as the Ultrasound Student Instructor Cadre (USSIC). This consists of second-year medical students (MS2s) who have completed the ultrasound curriculum as MS1s and who are interested in teaching ultrasound. USSIC applicants are required to complete both remote and hands-on training. By implementing such a curriculum in the first two years, we aim to give students ample time to strengthen their foundation in ultrasound, thus allowing them the opportunity to apply their skills through POCUS during their clinical rotations. One of the advantages of a student-led curriculum is that it promotes the development of both the teacher and the learner. Additionally, the peer-to-peer teaching model offsets the burden of recruiting multiple trained faculty members, allows for a smaller student to instructor ratio, and fosters leadership within the next generation of healthcare providers [6].

This study aims to assess if our student-led ultrasound education meets the ultrasound educational objectives and the satisfaction of the students with the curriculum. To do this, we developed assessments of student knowledge before and after the ultrasound instruction. Students also completed an end-of-year survey assessing the overall curriculum and each of the sessions. The results from this study provide insight into a student-led training model for incorporation at other institutions.

This study was previously presented as a poster at the American Academy of Emergency Medicine (AAEM) 26th Annual Scientific Assembly on April 20, 2020.

## Materials and methods

Study design and population

This prospective observational study was conducted at the University of California, Riverside School of Medicine during the 2018-2019 academic year. The University of California, Riverside Institutional Review Board exempted this study. All MS1s were eligible to participate in this study.

USSIC instructor training

USSIC instructor requirements totaled 20 hours of training (several USSIC instructors ultimately participated in over 50 training hours), in addition to completing the MS1 integrated ultrasound curriculum and remaining in good academic standing. Over the summer between MS1 and MS2, rising MS2s interested in becoming USSIC instructors were required to complete ultrasound practice and education, including supervised ultrasound practice, community outreach ultrasound education, and SonoSim (online platform for ultrasound training) modules before formally being chosen as a USSIC instructor. All USSIC instructors first participated in a seminar on teaching theory and then were divided into committees (Community Outreach, Undergraduate Education, Technology, Education, and Research) based on instructor interest. In addition to MS1 instruction, the USSIC instructors also provided ultrasound instruction to various entities (e.g., health fairs, premedical conferences, regional medical and physician assistant students, and resident physicians). As MS2s, USSIC instructors received additional ultrasound education in workshops and pre-instructional sessions guided by our Ultrasound Thread Director, an ultrasound fellowship-trained emergency medicine physician. These pre-instructional sessions allowed USSIC instructors to solidify a foundational knowledge and image acquisition skill necessary to instruct MS1s, as well as provide additional clinical context and ultrasound pathology review following a scaffolding educational model. Finally, MS2 USSIC instructors would provide an ultrasound review to the MS1s of the relevant anatomy associated with their Gross Anatomy curriculum.

USSIC curriculum and assessments

**Figure 1:**
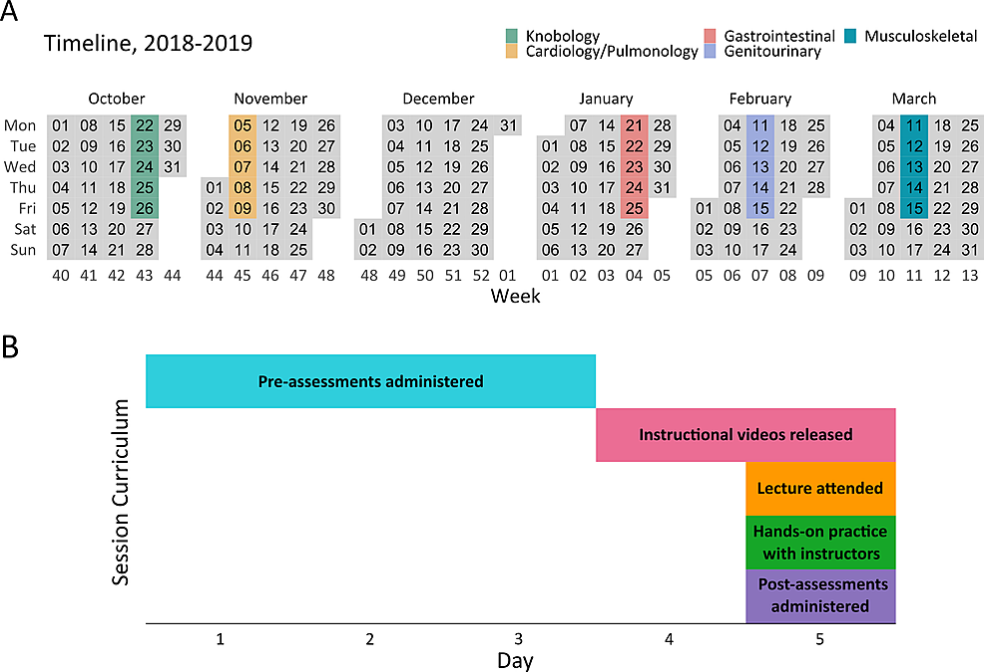
Curriculum timeline. A. Sessions were scheduled throughout the school year. B. Each session consisted of a five-day timeline. Pre-assessments were released before any educational exposure. Videos were released and first-year medical students reviewed them before the in-person lecture. Students then attended a didactic lecture followed by hands-on practice guided by Ultrasound Student Instructor Cadre instructors. At the end of their sessions, students took the post-assessment.

This study assessed the four teaching sessions derived from an established ultrasound curriculum created by the USSIC Education Committee: cardiopulmonary, gastrointestinal, genitourinary, and musculoskeletal. Each session consisted of a five-day timeline (Figures 1A, 1B). Prior to the core four teaching sessions, the MS1s underwent a knobology course that addressed how to use ultrasound machines, the physics behind ultrasound, and a basic understanding of artifacts that ultrasound can produce (Figure 1A). Before any ultrasound exposure related to the topic, MS1s received a link to complete a 15-minute pre-assessment. All assessments were distributed and timed through Qualtrics (Provo, Utah) [12]. Once the pre-assessment was closed, students were given a link to preview an ultrasound instructional video. Instructional videos were created for each of the four teaching sessions to provide MS1s with a preview of what techniques they would be utilizing during their session. The education committee created the scripts which summarized the instructional PowerPoint presentations while the accuracy of the information was sourced from expert ultrasound educators. Each video was composed of a sonographer describing the educational goals while demonstrating proper view acquisition on a USSIC model. Filming, editing, and directing were led by the USSIC student director and the technology committee.

During the ultrasound teaching session, a USSIC instructor gave a didactic lecture detailing the ultrasound views and relevant sonographic anatomy related to the theme of the session, paralleling the gross anatomy content theme. Additionally, a review of relevant uses of the scans and possible artifacts was taught. Afterward, groups of four to six MS1s dispersed into clinical suites for USSIC instructor-led hands-on probe experience and practiced acquiring views on standardized volunteer models. Our Ultrasound Thread Director rotated through each room to provide additional instruction as needed. After the teaching session, MS1s reconvened in the classroom and were given 15 minutes to complete the post-assessment (Figure 1B). Each pre-assessment and its corresponding post-assessment consisted of the same 9 to 10 multiple-choice questions tailored to meet the learning objectives created by the USSIC education committee to assess understanding of relevant sonographic anatomy, ultrasound technique, and other information presented during the teaching sessions. To meet content validity, the questions were derived from our learning objectives and instructional material and were approved by our Ultrasound Thread Director. To provide response process validity, MS1s were able to schedule an appointment for feedback after the post-assessment and supplemental learning opportunities. In terms of consequence validity, these assessments were for research purposes only, and results were not used to impact grades; however, the results were used to identify learning objectives that need to be more effectively taught during the USSIC-led teaching sessions.

End-of-year survey

An additional post-survey composed of 33 questions with three-point ordinal ratings (agree, neutral, and disagree) assessed the overall curriculum for how well the lessons corresponded with the MS1 didactic curriculum, their perceived preparedness for future clinical rotations, and their understanding of ultrasound technology and technique. It also assessed how confident students felt in acquiring specific views and in teaching other students how to obtain specific views.

Data analysis

Data were extracted from Qualtrics and analyzed with R, version 4.0.3 (R Core Team, Vienna, Austria) [13]. For the pre- and post-assessments, each correct answer was assigned one point with a maximum of 9 to 10 points possible per assessment. Normality was assessed with the Shapiro-Wilk test. Paired Wilcoxon tests were performed to assess the differences in the pre- and post-assessment scores for each of the four teaching sessions. Assessments were out of a maximum score of 100%. Student participation was variable; each assessment ranged between 54 and 70 participants. The end-of-year survey three-point ordinal ratings responses were converted to points: agree = 3, neutral = 2, and disagree = 1. Results were stratified between “USSIC” (n = 28; MS1s who became USSIC instructors as an MS2) and “non-USSIC” (n = 35; MS1s who opted out of becoming a USSIC instructor as an MS2) to assess if their perceived preparedness and overall satisfaction with the course had any correlation with whether they became USSIC instructors. Mann-Whitney U tests were performed between USSIC vs. non-USSIC students. Students used their unique student IDs to initially take the assessment and were subsequently de-identified. Duplicate and incomplete quizzes and surveys were omitted from the analysis. A *p*-value <0.05 was considered significant.

## Results

Up to 64 students were assessed among the different sessions. The pre- and post-assessment scores for MS1s are reported in Table 1. For each teaching session, MS1s performed significantly better on the post-assessment compared with the pre-assessment, after undergoing the MS1 ultrasound curriculum (*p* < 0.0001). There were no significant differences in the assessment scores between MS1s who became USSIC instructors and those who did not.

**Table 1:** First-year medical students’ pre- and post-assessment scores. Assessments were out of a maximum score of 100%. All values are mean ± standard deviation.

Session	Pre-assessment score (%)	Post-assessment score (%)	n	p
Cardiopulmonary	45.6 ± 16.9	82.9 ± 9.4	55	<0.0001
Gastrointestinal	53.9 ± 18.0	84.1 ± 13.5	54	<0.0001
Genitourinary	68.9 ± 19.1	91.4 ± 14.4	64	<0.0001
Musculoskeletal	33.6 ± 14.7	78.2 ± 18.2	55	<0.0001

To identify student satisfaction with various areas of the course, we distributed the end-of-year survey to all the MS1s. Responses with a mean score ≥2.50 indicate most students “agreed,” means between 1.50 and 2.49 indicate most students remained “neutral,” and means ≤1.49 indicate most students “disagreed” (Table 2). MS1s agreed with most of the survey questions with a few notable exceptions. Most students did not feel comfortable teaching the cardiopulmonary, gastrointestinal, genitourinary, and musculoskeletal exams, regardless of whether they became USSIC instructors.

**Table 2:** End-of-year survey responses. All values are mean ± standard deviation. Three-point ordinal ratings responses were converted to numerical points: agree = 3, neutral = 2, and disagree = 1. A mean score ≥2.5 indicates that most students “agreed,” between 1.5 and 2.49 indicates most students remained “neutral,” and ≤1.49 indicates most students “disagreed.” Abbreviations: USSIC, Ultrasound Student Instructor Cadre.

Question	USSIC (n = 28)	Non-USSIC (n = 35)	p
Overall			
Compared to the beginning of the year, I feel confident in my understanding of how ultrasound works.	2.9 ± 0.4	2.9 ± 0.3	0.59
I now feel enthusiastic about the use of point-of-care ultrasound.	3.0 ± 0.0	2.9 ± 0.4	0.07
I believe this ultrasound experience has prepared me for clinical rotations.	2.8 ± 0.5	2.2 ± 0.8	<0.001
I now have a better understanding of ultrasound applications across various fields of medicine.	2.9 ± 0.2	2.8 ± 0.5	0.14
I intend to continue learning about ultrasound and refining my skills.	3.0 ± 0.0	2.7 ± 0.6	<0.01
I felt that I had sufficient time to adequately master the learning objectives.	2.6 ± 0.6	2.0 ± 0.7	<0.01
I felt that the ultrasound videos and lecture slides were key to learning the material.	2.9 ± 0.2	2.8 ± 0.4	0.09
I would like ultrasound to grow at the University of California, Riverside School of Medicine, and be incorporated in other aspects of our medical education (e.g. Objective Structured Clinical Examination and clinical skills).	2.9 ± 0.3	2.5 ± 0.7	<0.01
The ultrasound instructors felt confident teaching their respective sessions.	2.9 ± 0.4	2.6 ± 0.7	0.08
The ultrasound instructors were open to questions and feedback.	3.0 ± 0.0	2.9 ± 0.2	0.20
The ultrasound instructors were knowledgeable in anatomy and physiology during their respective sessions.	2.9 ± 0.3	2.7 ± 0.6	0.13
The ultrasound instructors inspired me to become an instructor myself next year.	2.9 ± 0.3	2.5 ± 0.7	<0.01
The ultrasound instructor-to-student ratio was appropriate.	2.8 ± 0.5	2.9 ± 0.2	0.21
Cardiopulmonary			
I feel comfortable performing a cardiovascular scan on my own.	2.8 ± 0.5	2.2 ± 0.8	<0.01
I feel comfortable performing a pulmonary scan on my own.	2.6 ± 0.5	2.1 ± 0.8	0.03
I know how to apply proper probe techniques for the cardiovascular scan.	2.9 ± 0.3	2.6 ± 0.7	0.06
I know how to apply proper probe techniques for the pulmonary scan.	2.8 ± 0.4	2.5 ± 0.7	0.02
I feel comfortable teaching someone else the cardiovascular ultrasound exam.	2.6 ± 0.6	2.2 ± 0.8	0.09
I feel comfortable teaching someone else the pulmonary ultrasound exam.	2.4 ± 0.6	2.1 ± 0.8	0.16
The cardiovascular ultrasound session complemented my studies this block.	2.9 ± 0.3	2.8 ± 0.5	0.22
The pulmonary ultrasound session complemented my studies this block.	2.9 ± 0.3	2.7 ± 0.5	0.08
Gastrointestinal			
I feel comfortable performing a gastrointestinal scan on my own.	2.6 ± 0.6	2.4 ± 0.7	0.24
I know how to apply proper probe techniques for the gastrointestinal scan.	2.8 ± 0.4	2.7 ± 0.6	0.26
I feel comfortable teaching someone else the gastrointestinal ultrasound exam.	2.4 ± 0.6	2.2 ± 0.8	0.27
The gastrointestinal ultrasound session complemented my studies this block.	2.9 ± 0.4	2.8 ± 0.5	0.94
Genitourinary			
I feel comfortable performing a genitourinary scan on my own.	2.6 ± 0.6	2.4 ± 0.7	0.17
I know how to apply proper probe techniques for the genitourinary scan.	2.8 ± 0.4	2.6 ± 0.6	0.46
I feel comfortable teaching someone else the genitourinary ultrasound exam.	2.5 ± 0.7	2.3 ± 0.8	0.33
The genitourinary ultrasound session complemented my studies this block.	2.9 ± 0.4	2.8 ± 0.5	0.50
Musculoskeletal			
I feel comfortable performing a musculoskeletal scan on my own.	2.7 ± 0.6	2.4 ± 0.7	0.09
I know how to apply proper probe techniques for the musculoskeletal scan.	2.8 ± 0.4	2.7 ± 0.5	0.28
I feel comfortable teaching someone else the musculoskeletal ultrasound exam.	2.4 ± 0.7	2.4 ± 0.7	0.81
The musculoskeletal ultrasound session complemented my studies this block.	2.9 ± 0.4	2.7 ± 0.5	0.35

However, MS1s who became USSIC instructors as MS2s answered the survey significantly differently than the students who did not become USSIC instructors for the following: the ultrasound experience prepared them for clinical rotations, intended to continue learning and refining their skills, felt they had sufficient time to master the learning objectives, wanted to increase the presence of and incorporate ultrasound into the curriculum, and felt inspired by their USSIC instructors to become USSIC instructors themselves. Students who became USSIC instructors as MS2s also felt more comfortable independently performing pulmonary scans with proper probe techniques and cardiovascular scans compared to non-USSIC students. Overall, MS1s were neutral (2.3 ± 0.7, n = 63) regarding sufficient time to adequately master the learning objectives of the overall curriculum. However, once stratified, answers were significantly different; USSIC agreed (2.6 ± 0.6, n = 28) while non-USSIC remained neutral (2.0 ± 0.7, n = 35, *p* < 0.01). Overall, MS1s agreed (2.5 ± 0.7, n = 63) to believing that their ultrasound experience prepared them for their clinical rotations but once stratified the results were significantly different; USSIC agreed (2.8 ± 0.5, n = 28) while non-USSIC remained neutral (2.2 ± 0.8, n = 35, *p* < 0.01).

## Discussion

As in most fields of medicine, the advent of new technologies takes time to become integrated [2]. Yet, we must stress the benefits of even minimal ultrasound training. We believe that the MS1s who ultimately did not become USSIC instructors as MS2s will still carry their ultrasound knowledge into their clinical rotations. The importance of even minimal ultrasound exposure is underscored in a study by Kobal et al., which demonstrated the diagnostic accuracy of MS1s with brief echocardiographic training; MS1s were able to identify 75% of valvular disease, left ventricular dysfunction, enlargement, and hypertrophy using hand-carried ultrasound devices, while experienced board-certified cardiologists only identified 49% of the aforementioned pathologies after performing cardiac physical examinations [14]. In another study by Mouratev et al., medical students were more accurate in measuring liver size with ultrasound compared to board-certified internists measuring with a physical examination [15]. Since diagnostic accuracy is dependent on the operator’s skill, it is essential to train students during their undergraduate medical education so they employ these basic skills once they are in residency. It must be mentioned that the risk of misdiagnosis with ultrasound is high in the hands of inexperienced practitioners and may result in false-positive or false-negative findings that either lead to unnecessary testing or underdiagnoses [2]. For these reasons, the early introduction of ultrasound training is beneficial both for physicians and their patients.

Previous studies highlighting the need for ultrasound education in medical school curricula often use a faculty-led ultrasound instruction model; however, studies on effective student-led ultrasound instruction recommend a dynamic educational model [11,16-18]. USSIC training provides a standardized foundational understanding of ultrasound. The comparisons between the pre- and post-assessments indicate a statistically significant improvement after completing the ultrasound curriculum for each session, with the musculoskeletal session exhibiting the largest improvement. The results demonstrate that the USSIC program successfully taught the learning objectives. There was no significant difference in session assessment scores between MS1s who became USSIC instructors and those who did not, indicating that most students met the learning objectives regardless of their long-term interest in pursuing ultrasound. Based on the end-of-year survey, MS1s expressed an overall positive experience with the curriculum, with most students feeling confident in their knowledge of how ultrasound works and enthusiastic about using POCUS in the future. The results of our end-of-year survey mirror those of other medical schools that have also incorporated ultrasound in their curriculum [4,10,19]. Most students agreed that they could perform each sonographic exam independently; however, they felt neutral with the prospect of teaching the ultrasound techniques. This is congruent with our learning objectives because they did not have opportunities to teach ultrasound to their peers and for this reason, USSIC instructors must undergo rigorous training to acquire the necessary techniques to teach the learning objectives.

The majority of MS1s responded neutrally to feeling they had sufficient time to adequately master the learning objectives of the overall curriculum; however, once stratified, the responses were statistically significant with the majority of USSIC responding in agreement while non-USSIC remained neutral. This contrasts with MS1s mastery of the learning objectives, exhibited by improved assessment scores. Anatomical familiarity is another reason why students may have felt like they did not have sufficient time to adequately master the learning objectives [20]. Medical students often have difficulty translating 3D anatomy into 2D ultrasound views. However, in a study by Knudsen et al., it was identified that hands-on ultrasound training significantly improved long-term retention compared with hands-off training [20]. This provides insight into a potential source for the lack of confidence and comfort reported in the end-of-year survey. While there are drop-in hours allotted for MS1s to practice hands-on ultrasound, it was noted that few students used this resource because it was voluntary rather than a requirement. This is further support for the importance of integrating ultrasound into the curriculum.

It must be emphasized that our student-led ultrasound model does not seek to replace physician-led didactics, but rather aims at demonstrating the advantages of using a student-led ultrasound model [17]. The use of student instructors is cost-effective, propagates longitudinal learning through MS2s, and sets the foundation for a vertical model that may eventually expand to include MS3s and MS4s. Additionally, we believe this student-led model increases the level of ownership a student takes in their education when they choose to become an instructor.

A significant limitation of this study was the voluntary response bias, which was compounded by varying student participation for the pre-assessments relative to the post-assessments. This was mainly because students completed the pre-assessments at their discretion. There may have also been differences in the examination setting as we assume that students who took the pre-assessment did so in a testing environment comparable to the large, quiet lecture hall in which the post-assessments were administered. Lastly, although we would have preferred to provide a beginning-of-year survey at the start of the MS1 curriculum to compare with our end-of-year survey, the end-of-year survey questions were phrased to assess students’ perception of growth. Additionally, surveys to assess long-term retention of knowledge should be implemented at the post three- and six-month time periods. While similar studies identified that learners perceived student instruction to be comparable to that of faculty, future studies should determine whether students performed better on post-assessments after being randomized into cohorts taught by students and by physician faculty [16].

## Conclusions

This study illustrates how our student-led ultrasound curriculum was integrated into our institution’s education curriculum and quantifies the extent to which a student-led ultrasound curriculum enhances the MS1s understanding of ultrasound and sonographic anatomy. The pre- and post-assessments demonstrate significant improvements in student knowledge suggesting that there is value in integrating ultrasound in this manner. The end-of-year survey also suggests increased interest, confidence, and comfort in students who ultimately become USSIC instructors. By familiarizing students with ultrasound during medical school while simultaneously developing their ability to teach, we aim to better prepare them for their clinical rotations, medical residency, and future patient care.
